# “It's Never Too Early”: Preconception Care and Postgenomic Models of Life

**DOI:** 10.3389/fsoc.2020.00021

**Published:** 2020-04-21

**Authors:** Michelle Pentecost, Maurizio Meloni

**Affiliations:** ^1^Department of Global Health and Social Medicine, King's College London, London, United Kingdom; ^2^Department of Anthropology, University of Cape Town, Cape Town, South Africa; ^3^Alfred Deakin Institute for Citizenship and Globalization, Deakin University, Melbourne, VIC, Australia

**Keywords:** preconception, plasticity, epigenetics, DOHaD, Canguilhem, biopolitics, heredity

## Abstract

In this article, we are concerned with the expanded public health interest in the “preconception period” as a window of opportunity for intervention to improve long-term population health outcomes. While definitions of the “preconception period” remain vague, new classifications and categories of life are becoming formalized as biomedicine begins to conduct research on, and suggest intervention in, this undefined and potentially unlimited time before conception. In particular, we focus on the burgeoning epidemiological interest in epigenetics and Developmental Origins of Health and Disease (DOHaD) research as simultaneously a theoretical spyglass into postgenomic biology and a catalyst toward a public health focus on preconception care. We historicize the notion that there are long-term implications of parental behaviors before conception, illustrating how, as Han and Das have noted, “newness comes to be embedded in older forms even as it transforms them” (Han and Das, 2015, p. 2). We then consider how DOHaD frameworks justify a number of fragmented claims about preconception by making novel evidentiary assertions. Engaging with the philosophy of Georges Canguilhem, we examine the relationship between reproductive risk and revised understandings of biological permeability, and discuss some of the epistemic and political implications of emerging claims in postgenomics.

## Introduction

In 2018, the highly respected medical journal *The Lancet* launched its series on preconception health. The series editorial, titled “Campaigning for preconception health,” calls for “special attention to an underappreciated period in the lifecycle with far-reaching consequences across the lifecourse” (Lancet, 2018: p. 1,749). The series' conceptual framework and rationale for a renewed focus on preconception is based on Developmental Origins of Health and Disease (DOHaD) theory, which postulates that environmental factors in critical windows of development shape health and developmental outcomes, possibly via epigenetic mechanisms (Godfrey et al., 2010). The series comprises three papers. The first proposes an expanded definition of the preconception period along three perspectives: the biological perspective, focused on the “days to weeks” before conception; the individual perspective, referring to the intentionality of a couple to conceive; and the public health perspective, focused on the “longer periods of months or years to address preconception risk factors, such as diet and obesity” (Stephenson et al., 2018, p. 1,830). This third perspective is a significant departure point from previous definitions of the preconception window. It includes a major shift from ensuring “optimal intra-uterine conditions for the developing fetus” (Shannon et al., 2014), to defining preconception health as the key determinant of “next generation health” (Stephenson et al., 2018, p. 1,830). The second paper in the series outlines four mechanisms of “periconceptional developmental conditioning,” via maternal overnutrition, maternal undernutrition, paternal effects, and the use of assisted reproductive technologies (Fleming et al., 2018). The authors conclude that “the evidence for periconceptional effects on lifetime health is now so compelling that it calls for new guidance on parental preparation for pregnancy, beginning before conception, to protect the health of offspring” (Fleming et al., 2018, p. 1,842). The final paper in the series discusses possible interventions. In addition to food fortification, prenatal supplementation, and behavior change strategies that have been part of preconception care for some time, the authors call for the “creation of a social movement” that would involve entire populations and global coalitions to prioritize the periconceptional period (including pregnancy and preconception) as an important “teachable moment” for changes in diet and lifestyle (Barker et al., 2016, p. 330; Barker et al., 2018, p. 1,860). In a follow-up article to the *Lancet* series, recommendations focused on preconception health in England include a proposal for measuring core metrics for annual reporting on the state of preconception health (Stephenson et al., 2018). The paper's conceptual framework incorporates “the mechanisms whereby parental preconception exposures contribute to the developmental origins of health and disease” and recommends both public health and individual interventions. At the individual level, the recommendation is for a “reorientation of the health services and health-care professionals to *normalize* conversations about planning for pregnancy during routine visits” (our emphasis). The measurement of core metrics, as a set of preconception health indicators, is framed as a means for the state and relevant organizations to account for the improvement of preconception health (Stephenson et al., 2019).

The *Lancet* series exemplifies a renewed interest in “preconception care,” based on DOHaD theory. For example, in the UK and Australia, where the authors are, respectively based, recent guidelines on preconception care reference developmental origins as a key reason for focusing on the preconception period as part of public health strategy. Public Health England's “Making the case for preconception care” explicitly adopts the *Lancet's* individual/population approach to preconception and cites the possible intergenerational transmission of overweight and obesity (Public Health England, 2018), while practitioner guidelines in Australia cite fetal programming as a critical factor for why the preconception period is important (Dorney and Black, 2018).

In this article, we are concerned with this expanded public health interest in the “preconception period” as a window of opportunity for intervention to improve long-term population health outcomes. In particular, we historicize the notion that there are long-term implications of parental behaviors before conception; we consider how DOHaD frameworks justify a number of fragmented claims about preconception by making novel evidentiary assertions; and examine how DOHaD-inspired preconception campaigns may be shifting and expanding ideas of pre-conception risk and care. It is our contention that campaigns that seek to expand preconception interventions to include the general population raise important questions about what constitutes “health” in such frameworks, and how that health might be measured. In particular, we are interested in how, as Han and Das note, “newness comes to be embedded in older forms even as it transforms them” Han and Das (2015, p. 2). Engaging with the philosophy of Georges Canguilhem, we are concerned with the relationship between reproductive risk and revised understandings of biological permeability heralded by molecular biology and biotechnologies, and how this might re-inscribe the normal and the pathological, and enforce new norms and practices about normalization and normativity. As Rose noted in a 1998 essay on reading Canguilhem in the context of new genetics, “The judgements of probabilities and of risks that have become central both to experimental and clinical practice inescapably connect to the judgements of value that are placed upon different forms of existence and the logics of treatment they mandate” (Rose, 1998, p. 165). He poses the question: “What is normality at the level of our genetic code?” It would seem to us that a re-reading of Canguilhem today poses the question anew—what is normality given new understandings of development as an interplay between genetic and epigenetic factors and the power of the milieu? How do new findings blur the boundaries between the body as “at once a given and a product”? (Canguilhem, 2008, p. 472). How does this shift the binary between congenital and developmental views of health, i.e., health “as the state of the given body” and as “the expression of the produced body” (ibid, 473–474)?

This article builds on important scholarship, mostly focused on the United States, that has shown how frameworks of preconception care based on DOHaD expand the biomedicalisation of women's bodies; assume heteronormative definitions for sex, family, and care; exclude men from research and interventions; exhort women to engage in “anticipatory motherhood” (Waggoner, 2013, p. 347); and risk portraying motherhood as “the default social and clinical strategy in women's health care” (Waggoner, 2013, p. 366; Almeling and Waggoner, 2013; Gentile, 2013; Thompson et al., 2017). Our analysis proceeds along three lines of enquiry. First, we contextualize the DOHaD-inspired focus on preconception within a longer history of debates, controversies and ideas about the power of parents and ancestral events to alter heredity before and after conception. We highlight how the emergence of genetics in the twentieth century both limited and reframed this blurred view of heredity and generation, and its impact on ideas of antenatal intervention and responsibility. Rather than a genealogy of preconception care, this section offers an overview of how the concept of “preconception care” ties to historically shifting views about the nature of heredity and reproduction. Second, we consider how DOHaD theory informs the scientific literature on preconception interventions and how this constitutes a new assemblage of approaches to evidentiary knowledge production, prognostication, and practice. Here we build on scholarship that has questioned the evidence base for preconception intervention (Waggoner, 2013, 2017), turning the lens on how DOHaD-informed justifications for intervention before pregnancy are instituting new norms and practices. As Miranda Waggoner has shown for the US, preconception care models predate the postgenomic fixation on maternal bodies as key sites of intervention. This is certainly also the case elsewhere, but we will argue that the more explicit adoption of DOHaD frameworks in preconception care, which the *Lancet* series exemplifies, marks an important shift in terms of the kinds of evidence used and the categories now formalized in these interventions.

The *Lancet* series discusses the difficulties of defining the preconception period, given that “a time period before conception can only be identified after a woman has become pregnant” (Stephenson et al., 2018, p. 1836). Three months prior to conception has been a standard reference point, though some have called for broadening this to 1 year (Dean et al., 2013). We argue that, despite definitions of the “preconception period” remaining vague, these discussions are nevertheless formalizing new classifications and categories of life as legitimate sites for research and intervention for the health of future generations. Third, we draw on Canguilhem's concepts of the normal and the pathological, and the living and milieu, to consider the new norms instituted by a return to a focus on “preconception health,” and we consider the implications of these shifts for gender and reproductive risk, and approaches to individual and population health.

## Preconception Before And After The Rise Of Genetics

The notion that parental behavior and experiences before or during conception shape the physiology of future generations is far from new. Indeed, in Canguilhem's own explorations of the histories of “normal” and “pathological” states, he discusses interpretations of the differences between Chinese and European urinary outputs as an effect of diet and differences in the “nutritive rhythms determined by ancestral experience” (Canguilhem, 1991, p. 167). Canguilhem also cites 1930s' French medical theory on the etiology of nutritional diseases as “diseases created by the *soft upbringing* provided by parents…above all of bad habits of life and diet which individuals must avoid and which parents already afflicted with nutritional disturbances must avoid passing on to their children” (Canguilhem, 1991, p. 169, italics in original).

The concept of intergenerational transmission of traits (for DOHaD, the transmission of risk) predates and has coexisted with modern biomedicine. What we would call today the malleability of heredity, corporeal plasticity and the power of the parental milieu are ubiquitous in ancient humoral views of the body, early modern European medicine, and Ayurvedic medicine, among others (Lock, 1993; Langford, 2002; Anderson, 2016; Meloni, 2019). One case in point is telegony—literally “generation at distance.” Widely influential in animal husbandry in Europe and North America until the early twentieth century, telegony posits that offspring will inherit the characteristics of a *previous* sexual mate of the mother, not their immediate biological father (Burkhardt, 1979; Bynum, 2002a). When applied to human reproduction, telegony became the basis for a number of conflicting claims about fears of racial mixing, the long-term effects of rape atrocities in war (Harris, 1993), and the potential tainting of offspring born to a mother with multiple sexual partners (a notion currently revived in Orthodox Russia: Sudakov, 2007). The acceptance of notions like telegony is indicative of the importance of theories of biological memory in evolutionary debates up to the early twentieth century (Bowler, 2003). Generation and heredity in this frame were porous to multiple influences, and this was often the basis for placing demands on women, conventionally understood in ancient and early modern medicine as of a softer and more permeable nature (Dean-Jones, 1994; King, 1998).

While classical sociological analyses have tended to emphasize the invasive impact of early-twentieth-century medicine in regulating the maternal body (Oakley, 1984), this modernist view may overlook the heavy moralizing burden of premodern views of generation for women (Kukla, 2005; Meloni, 2019). The best example is the notion of *maternal impression*—the capacity of a woman to mark, imprint or deform the fetus through “imagination”—understood as a key mechanism of heredity and generation until the rise of genetics. Examples of maternal impressions as a result of observing scary events, objects, statues and people (often racialized figures) were widespread in ancient humoralism and early modern medicine in Europe. Albeit in a context where the experience of pregnancy remained mostly private and non-medicalized, anxieties about the emotional states of mothers-to-be were part of wider recommendations by midwives and doctors about the emotions, lifestyle and mental states of mothers. This is not the biopolitics that we have known since the rise of the disciplinary mechanisms of modernity. Biopower does not map perfectly onto the rise of modern biomedicine and it is fair to recognize that the link between health and normality and the patriarchal impact of the medical gaze, *pace* Foucault (1973, p. 35), is older and possibly even more pervasive in traditions preceding modern medicine. In the biopolitics of these premodern medical writings, notions like corporeal imbalance blurred together disease and moral transgression, and dietetic recommendations have to be understood as both about health and moral corruption. The government of female biology before the rise of biomedicine has to be understood in this highly moralized context. To make sense of a female body always considered of a softer nature, technologies of body control were deployed through the existence of cultural fears, common sense, and shared biases particularly around the experience of pregnancy. At the peak of more than 100 years of medical and midwifery texts containing harsh prescriptions on the behavior of pregnant women, we can read in John Maubray's influential *The Female Physician* (Maubray, 1724) that during pregnancy women should “suppress all Anger, Passion, and other Perturbations of Mind, and avoid entertaining too serious or melancholick Thoughts; since all such tend to impress a Depravity of Nature upon the Infant's Mind, and Deformity on its Body” (cited in Shildrick, 2001, p. 42). Although the concept of maternal impressions is focused on the pregnant woman rather than the preconception period, it is worth mentioning here because it reveals not only a longer history of normalization technologies, but also the deep historical asymmetry in debates on maternal effects in favor of the transmission of defects rather than of positive traits (Meloni and Müller, 2018). Historically, this emphasis on the corrupting womb became even more visible in the popular imagination with the rise of Protestantism in Northern Europe (Fissell, 2004), which led to a darker association of the power of the womb with ideas of danger and risk (rather than with positive nurturing, as in the image of Mary). Once again the combination of health and normality is older than suggested by authors who superimpose biopolitics onto eighteenth or nineteenth century modernity.

While views of maternal impression were still popular among doctors and the public until the nineteenth century, it is only at the end of the nineteenth century that ideas of “pre-natal culture” became institutionalized in medicine through fields like fetal physiology and antenatal pathology (Arni, 2015, p. 2016). Caroline Arni's work on the German and French medical context, for instance, has highlighted the emergence of these disciplines around ideas of “trans-natal continuity” and intergenerational transmission that “confronts developmental continuity with historical contingency” Arni (2015). This emphasis on the potential hereditary morbidity of historical events is visible in the work of Charcot's disciple Charles Féré. Arni (2016) notes how the traumatic events of 1870–1871 in Paris (defeat by Prussian troops and later the instauration of the commune) gave rise in France to medical debates about possible moral and mental shocks in mothers and the effects on their unborn children conceived during the political turmoil. The “enfants du siege” (children of the siege), as defined by Féré himself, became a “synonym of ill formed and doomed children” (Féré, 1884, p. 245, our translation) as a result of “emotional states of the mother that (…) translated into nutritive disturbances and vascular contractions” (Arni, 2016, p. 294). Besides widespread notions of degeneration and inheritance of parental disease (Cartron, 2007), France was also the site for ideas and practices of *puericulture*, which included extended attention to the mother's health during pregnancy and to both parents' physical condition *before* marriage (Schneider, 1982). “Pre-maternity ward” clinics and “ante-natal therapeutics” were proposed at the turn of the twentieth century in Scotland by John William Ballantyne, one of the founders of perinatal pathology, as an environmentally based strategy (that is, alternative to eugenics) toward “race betterment” (Seigel, 2013).

Before their crystallization around ideas of unchanging genes, early twentieth-century eugenic debates often reflected ideas about transmission of “defects to subsequent generations' (Stockard, 1913) or racial poisoning (mostly via parental alcoholism: see Saleeby, 1914), as well as more optimistic views on the possibility to alter the course of morbid heredity via prenatal intervention. For geneticists and mainstream eugenicists however, these ideas were shocking. A vitriolic article in 1915 in the flagship genetics journal, *Journal of Heredity*, criticizes some wings of the eugenic movements (branded in the article as “prenatal culturists” and “maternal impressionists”) because they still indulged in the notion that good mothers could “overcome the effect of heredity” by using “self-control, cheerfulness, [and] love”. For the unsigned editor of the journal, the idea of “improv[ing] the race on a large scale, by the general adoption of pre-natal culture” was just a “short cut” to eugenics, and a “superstitious” one (Journal of Heredity editorial, 1915, p. 512, 516). “It should be a satisfaction to mothers to know,” the article continued, “that their children will not be marked or injured by untoward events in the ante-natal days; that if the child's heredity cannot be changed for the better, neither can it be changed for the worse”. The attack on the “airy fabric of pre-natal culture” and the reassurance to mothers that they could do limited harm illustrates the ambiguous moral landscape of ideas of maternal impression and prenatal culture. The long-term prevalence of these views, for instance in the preformation–epigenesis debate, put women in a paradoxical situation with regard to the normal and the pathological (Bynum, 2002b). It gave mothers a certain active power to shape the physiology of the fetus through a number of both positive (attending concerts or literary talks during pregnancy, as in nineteenth-century popular culture, or just looking at the portrait of a beautiful child) or negative actions (refraining from sad thoughts or vivid emotions), while at the same time placing on them a heavy responsibility and rigid demands of their behavior demands (Epstein, 1995).

The rise of genetics had a disruptive effect on this highly moralized landscape (Meloni, 2019). Genetics consolidated its own evidentiary terrain on heredity, producing forms of risk, prudence and obligation for governing the conduct of the genetically at-risk person or family (Rapp, 2000; Rose, 2007). However, in reducing mothers to the role of passive carriers of a set of genetic instructions for the next generation and arguing that maternal behaviors (with few exceptions) had no direct effect on patterns and pathologies of chromosomes (Rapp, 2000), genetics significantly reconfigured the space of parental responsibility. As we can read in a 1930 popular American publication about pregnancy, *Parents' Magazine*: “No mother can any longer think of herself as overwhelmed by the task of making her child; she must regard herself as the trustee of something far finer than she could possibly make single-handed. This change in point of view means that while the mother can no longer hope to produce a preacher by reading sermons she need no longer fear that if frightened by a mouse or what not she will deposit a ‘birthmark' in the shape of a mouse upon the child” (cited in Oaks, 2001, p. 21).

This does not mean of course that genetics and wider technologies connected to genetic science (such as assisted reproductive technologies) have entirely erased an interest in the social characteristics of the “trustees” or carriers of genetic material, such as sperm and egg donors. As in all complex and multilayered social phenomena, tensions do exist between the conceptual aspects and societal and cultural uptake of a discipline like genetics. For instance, social or educational characteristics of sperm or ova donors are still an important piece of information, but mostly as a “re-enchantment mechanism” to counterbalance anonymity, de-commodify the donation, or generally reassure prospective parents about the good quality of the reproductive material (Bokek-Cohen and Gonen, 2015). However, it is because the genetic material is highly abstracted from its surroundings, and its carriers' biography, that this supplemental personalization is required. Intimacy is constructed as a *simulacrum* to fill the gap between a genotype and the advertised identity of the phenotype (ibid.). This connection between genetic and social knowledge is quite dissimilar from the pre- and post-genomic concern about direct environmental effects on the reproductive material, i.e., the establishment of a straight causal arrow from the parental phenotype (or its wider milieu) to the *next generation's* genotype. If we look at the longer history of this idea, the field of genetics successfully overturned it. Still framed with anxiety and guilt (for instance in relation to the age of pregnancy), notions of the normal and the pathological for mothers shifted significantly from an overall behavioral concern about proper action to protect the unborn, to a strictly biomedical categorization of innate risk.

What society could read into reproductive abnormality was significantly altered by the notion of a discrete and unchanging gene. Given the fundamental randomness of DNA mutations and the insulation of DNA from parental environments, genetic disease to a significant extent did not imply good or bad parental behavior or lifestyle (Meloni, 2019). Rather, responsibility was reframed as a duty *to know* the content of one's hereditary script (using the body as a window into this script), and to act on this knowledge through medical rather than behavioral or lifestyle intervention (Meloni, 2019). A decision to terminate pregnancy, or avoid pregnancy at all, could follow. It is important however to remember that for the large majority of cases, genetic testing ends with reassurance for parents as carriers of a risk-free pregnancy. Moreover, the same ideas of intensive screening for chromosome abnormalities sustained a utopian view of medical control on the developmental outcome of the fetus (Rosenberg, 2007) that facilitated “the management of uncertainty” (Löwy, 2014, p. 160). As historian Ilana Löwy highlights, “learning that that their child's problems are ‘genetic' can alleviate the parents' guilt: the child's condition was an unforeseeable accident and not, for example, the consequence of a mother's behavior during her pregnancy” (Löwy, 2014, p. 159).

This departs from older frameworks, discussed above, in which the moral responsibility of mothers (for instance excessive sexual desires, food cravings or abnormal conduct) or their vulnerability to traumatic events was linked directly to visible physiological effects on the child. We are not claiming here that we are witnessing a return of the old maternal impression framework as such, or that the current postgenomic scenario is entirely transformative of the logic of genetic determinism. Firstly, many of the molecular mechanisms that constitute the evidentiary terrain of epigenetics (and partly DOHaD) do not represent a clear-cut move away from the language and rhetoric of gene power (Waggoner and Uller, 2015). Secondly, and more importantly, the history of science is less about clear-cut breaks or a return of the identical than a complex process of bricolage of old and new through “processes of maintenance that require explanation no less than transformation do” (Arni, 2016, p. 286)^1^.

While we do not claim that an extended view of biological heredity in contemporary epigenetic frameworks *necessarily* reinvigorates this highly moralized landscape, we suggest that the shift from gene centrism to wider environmental and lifestyle factors is not inherently liberating for parents or parents to be, and can instead compel new behaviors and forms of discipline and responsibility.

Preconception care as a concept is thus not entirely new, and the focus on women's lives prior to their pregnancies has taken on different forms. The contemporary definition of preconception care is “the provision of healthcare to women of reproductive age and their partners prior to conception in order to optimize a woman's physical, social and emotional well-being and to ensure optimal intra-uterine conditions for the developing fetus” (Shannon et al., 2014). “Early life” is increasingly broadly defined, such that epigenetic effects on health trajectories are thought to be possible not only during the *in utero* period but even prior to conception in an anticipatory mode of a “folded futurity” (Mansfield, 2017).

In sum, the call for a renewed focus on preconception health is best understood as part of a longer history of ideas about heredity. The incursion of public health further and further into an ever-expanding “preconception” window—in the extreme case, starting at an individual's birth—finds important historical resonances in changing notions about the nature of biological heredity, the innate and the acquired (Bonduriansky and Day, 2018). In the twenty-first century, genetic determinism has been replaced by “developmental programming,” “genomic imprinting,” and “epigenetic memories” (Reik and Walter, 1998; Cottrell and Ozanne, 2007; Thayer and Kuzawa, 2011). Plasticity, rather than fixedness, has become a key lens through which development is understood, and epigenetic imprinting is now thought to occur in critical windows of human development—early life, adolescence, preconception, pregnancy, and, in principle, over the whole life-course—to impact offspring biology (Bateson and Gluckman, 2011; Moore, 2015, p. 168 and ff.). In spite of being relatively recent in its evidentiary consolidation, this has a significant impact on how reproductive risk, intervention and responsibility is understood. We turn now to the DOHaD evidence base for preconception care to consider how this field is defining new categories for intervention.

## New Kinds Of Evidence For Preconception Intervention? Some Model Problems

In her in-depth analysis of preconception care in the United States, Waggoner shows how preconception interventions have been a part of women's health programs since the 1980s Waggoner (2013). While sympathetic to public health attention to women's health, Waggoner shows how the “conflation of women's health and maternal health” to formalize preconception care is based on quite limited evidence (Waggoner, 2013, 2017, p. 346). What does DOHaD research mean for this assertion?

First, it is important to recapitulate the paradigm shift in epidemiology at the end of the twentieth century that has found resonance in a peculiar articulation of DOHaD and the fetal origins hypothesis^2^. The term “life course epidemiology” first appeared in 1997; since that time, this has been formalized as an important field and generated a range of conceptual frameworks and methodologies to understand relationships between exposures and outcomes across the life course. While DOHaD and epigenetics researchers study developmental mechanisms, life course epidemiologists are concerned with “pathways” and determining the best timing for interventions (Ben-Shlomo et al., 2016). Life course epidemiology was proposed as a new model for studying chronic disease, based on the fetal origins hypothesis that is the basis for the DOHaD field (Kuh and Ben-Shlomo, 1997). In practice, for prominent scientists in the DOHaD field, DOHaD, epigenetics and life course epidemiology are triangulated in a complementary framework that has fundamentally altered public health approaches to chronic disease (Gluckman et al., 2016). A life course approach articulates different phases of life and distinct critical or sensitive periods. For example, Ben-Shlomo et al. (2016) delineate six phases: (pre)conception, pre-natal, pre-pubertal, pubertal, maturity, and senescence. As Lappé and Landecker (2015, p. 152) have argued, the genome now also has a life span, which “aligns the molecular and the experiential in new ways, shifting ideas of life stages, their interrelations, and the temporality of health and disease.” While studies of “development” might have been previously confined to the fetus, infant or child, “early life” is now a far more fungible category that includes the “periconceptual” and “preconceptual” (ibid, p. 160). In the same way that “the maternal environment” has expanded its definition to become a much broader spatio-temporal concept than before, so too has the “fetus” grown to encompass the “phantom fetus” or “preconception fetus” that exists as a potential future to be accounted for through action in the present (Waggoner, 2013; Richardson, 2015; Lock and Palsson, 2016; Mansfield, 2017).

DOHaD and life course approaches further broaden the preconception period to include life stages considered as “sensitive” periods for establishing health behaviors. For example, a 2014 editorial in *Reproductive Health* calls for preconception health to include “the welfare of children, reproductive aged individuals, and those transitioning between these periods” (Mumford et al., 2014, p. 2). A 2016 review highlights conception and the days following it as a crucial time for nutritional support during early embryogenesis, and concludes that “the time has arrived to begin parenting before conception” (King, 2016, see also Lane et al., 2014). The *Lancet* series focuses the “public health perspective” of preconception care for men and women on “a sensitive phase in the life course, such as adolescence, when health behaviors affecting diet, exercise, and obesity, along with smoking and drinking, become established before the first pregnancy” (Stephenson et al., 2018, p. 1,836). This expanded definition of preconception relates to the increased temporal distance between exposure and outcome that DOHaD, epigenetics, and life course epidemiology propose (Kuh and Ben-Shlomo, 1997). The *Lancet* series summarizes this important shift in how exposures and outcomes are now defined: “The relationship of exposures to outcomes can be considered in terms of critical periods, sensitive periods, and cumulative effects” (Stephenson et al., 2018, p. 1830). The previously sparse evidence base for preconception intervention expands significantly with its new definition. Birth cohort and longitudinal study designs are key tools in this field for testing hypotheses and producing evidence of associations between exposures and outcomes. Proving disease causation is no longer the central aim, rather, life course epidemiologists are concerned with the “multi-faceted traits and longitudinal trajectories of functional phenotypes that can be assessed well before any clinical threshold is reached” (Ben-Shlomo et al., 2016). Evidence in this case is derived from large longitudinal cohorts that can demonstrate associations that are statistically significant but can neither demonstrate causation nor confirm the mechanisms of these associations.

While DOHaD and life course theory are thus supported by an impressive corpus of observational and cohort studies, the scientific underpinnings of observed associations are still the subject of debate. The field of epigenetics has provided a set of possible mechanisms whereby environmental influences might impact gene expression, including DNA methylation, histone modification and DNA protein binding (Waterland and Michels, 2007). While an epigenetic basis for DOHaD associations is supported by an extensive animal model literature (reviewed by Gluckman et al., 2007), there is limited molecular evidence in human cohorts (Heijmans et al., 2008; Tobi et al., 2009; Waterland et al., 2010; Godfrey et al., 2011; Borghol et al., 2012, p. 63). The merging of DOHaD research and epigenetics has given rise to an “epigenetic epidemiology,” which remains however a large umbrella. Some of this research emphasizes processes of genomic and metabolic imprinting in development and metastable epi-alleles (Waterland and Michels, 2007); other research is instead framed within a life course perspective (Stringhini and Vineis, 2018). Significant challenges remain in articulating the biological mechanisms and pathways responsible for observed associations.

Take for example folate supplementation, perhaps the most widely accepted preconception intervention to date. An association between neural tube defects (embryonic malformations resulting in birth defects including spina bifida and anencephaly) and maternal folate deficiency was first hypothesized in the 1960s, and subsequently demonstrated in a number of studies including a large-scale randomized controlled trial by the British Medical Research Council (MRC Vitamin Study Research Group, 1991). Since the early 1990s, the World Health Organization has thus recommended folate supplementation for women trying to conceive, to be continued until the end of the first trimester (WHO and FAO, 2004). Since 2006, the WHO has also recommended food fortification to boost population folate intake (Crider et al., 2011). In the past decade, scientific efforts have turned to focus on the role of folate in DNA methylation, postulated to be the key epigenetic mechanism at work. As stated in a 2011 review article in the journal *Nutrients*, “There are a number of knowledge gaps that need to be filled before we know if folic acid affects disease risk through DNA methylation in humans. The ‘normal' patterns of DNA methylation across the genome are unknown, as are the ‘normal' levels of variation among and between individuals and populations” (Crider et al., 2011, p. 375). The authors note the evidence in animal models, and the current lack of evidence in humans.

Another example is fetal alcohol spectrum disorder (FASD). Researchers are only beginning to describe the molecular mechanisms of alcohol's teratogenic effects using animal models (Ungerer et al., 2013). Epigenetic mechanisms, again related to folate metabolism, are considered most likely, but are difficult to study, as they are transient and cell-specific (Ungerer et al., 2013). While these factors might be controlled for in mouse models, it is difficult or impossible to study these mechanisms in human models. A recent study compares DNA methylation patterns in prenatal-alcohol-exposed adult mouse brains with buccal swabs from human children with FASD (Laufer et al., 2015). What these two examples illustrate is the difficulty with which mechanisms of disease might be studied in human populations. Yet scientists' cautious translation of findings in animal models has been frequently sensationalized in media and popular discourse.

Perhaps most contentious in epigenetic research are notions of inter- and transgenerational epigenetic inheritance. While developmental programming refers to the establishment of long-term developmental effect *in utero* or early life, and is thought to be a key mechanism underpinning associations between early life exposures and adult health outcomes (Bianco-Miotto et al., 2017), inter- and above all, transgenerational effects are more heavily debated. Intergenerational epigenetic inheritance refers to the transmission of phenotypic changes to the fetus's progeny (mostly via *in utero* effects on the progenitors of germ cells), whereas transgenerational effects are the supposed transmission of phenotypic changes in the *third* female (F3) or second male (F2) generation of *unexposed* individuals (Skinner et al., 2013; Heard and Martienssen, 2014). A number of caveats have been raised regarding these concepts. While effects on the second generation are now documented in humans (Painter et al., 2008), observations of intergenerational patterns have often been ascribed to the persistence of environmental exposures and their effects on maternal physiology (Drake and Liu, 2010, p. 207). On the contrary, transgenerational epigenetic effects are well-documented in plants, fruit flies and nematodes, but are the subject of much controversy in mammals and humans (Heard and Martienssen, 2014; Horsthemke, 2018). As Marsit (2015) states, it is “difficult if not impossible to conclusively demonstrate the existence of transgenerational inheritance in humans, where control or at least adequate assessment of the environments experienced over three generations is not feasible.” Despite the caution with which scientists have posited theories of transgenerational epigenetic inheritance, the notion that “you are what your grandmother ate” has been widely popularized (Richardson, 2014).

Another key issue relates to the limitations of traditional epidemiological approaches for inferring causality in observational studies, a much-used study design in epigenetic epidemiology and DOHaD research (Heijmans and Mill, 2012). These include the instability of epigenetic mutations (unlike the genome), selection bias, confounding and reverse causation, and, while a number of new methods have been developed to try to improve causal inference in this research, the use of these techniques and how best to triangulate them is still work in progress (Gage et al., 2016). Of particular interest to us is the relatively little attention paid to the possibility of confounding due to heritable genetic variation (O'Donnell and Meaney, 2016). This is in keeping with the historical swing back to focusing on environmental and maternal behavioral factors (now framed as epigenetic) discussed in the previous section.

Finally, the DOHaD evidence base on which new calls for preconception care interventions are premised, is largely focused on maternal effects. Women have long been the focus of preconception care, despite evidence that men are also at risk of exposures that affect fertility (Guerrero-Bosagna and Skinner, 2014; see e.g., Carré et al., 2017). This is an effect of what Daniels (2006, p. 6) describes as “reproductive masculinity,” where men are positioned as distantly placed from reproduction and less susceptible to harm, privileging an image of resilient virility. In biomedical terms, genetic risk in the gene-centric view was figured as a “50/50 equation” between men and women, marking conception as a key moment of paternal contribution: “father as sperm” (Chiapperino and Panese, 2018, p. 1,233; see also Almeling and Waggoner, 2013). While it is true that for most DOHaD studies, mothers remain the main target of research (but see Soubry, 2018), significant work is focusing on the paternal germline. This includes the Överkalix cohort work, which emphasizes male-line transgenerational effects of food availability on longevity (Kaati et al., 2002). Other more recent studies have highlighted the effects of paternal obesity, fasting, overnutrition and low protein diet on offspring methylation, with implications for metabolic alterations and longevity (Carone et al., 2010; Ng et al., 2010; Rando and Simmons, 2015); the impact of preadolescent smoking on the sperm quality of first- and second-generation offspring (Soubry et al., 2013); and the effects of paternal stress on offspring behavioral and metabolic responses (Gapp et al., 2014a,b) and hypothalamic–pituitary–adrenal stress axis regulation (Rodgers et al., 2013). Evidence of the epigenetic effects of fathers' experiences, particularly of stress (Rodgers et al., 2013), could well-expand the preconception mandate, such that all humans of reproductive age are now entreated to act for the sake of the future (Kotelchuck and Lu, 2017).

It remains to be seen whether critical epigenetic reprogramming periods similar to pregnancy will be identified in men, but it is obvious that this range of studies on the sperm epigenome as “a messenger of ancestral exposures” (Soubry, 2015) have wide potential to expand ideas of preconceptional healthcare for fathers-to-be as well. In the 2018 *Lancet* series on preconception, Fleming et al. discuss the “paternal origin of periconceptional developmental programming” in some detail, noting the very limited evidence available, and pointing to the potential combination of maternal and paternal effects impacting on periconceptional development. As Chiapperino and Panese argue, the limited knowledge on paternal influences may lie “in the ways biological experiments can study parental care, and consequently produce discursive resources to know and norm this issue in our societies” (Chiapperino and Panese, 2018, p. 1,237). What has so far been a relative lack of incorporation into medical frameworks of paternal effects and an exaggerated focus on pregnant or pre-pregnant mothers may simply reflect an unconscious bias toward maternal effects that sidelines alternative approaches (Pentecost, 2018).

In sum, the DOHaD research that is currently founding a renewed investment in preconception care (with a continued focus on women) represents a novel evidence base that, while compelling, contains a number of challenges for the field concerning underlying mechanisms, causal inference, and the relative inattention so far to genetic variation and paternal factors. As Aagard-Hansen and colleagues concede, arguments for preconception interventions are currently “compelling but not well-researched” (Aagaard-Hansen et al., 2019). It is important to note that, given the difficulties described for studying mechanisms in human populations, the inadequacy of older statistical methods, and the introduction of study designs that integrate genomics, what counts as evidence is under renewed contestation (see also Bauer, 2013). As Martyn Pickersgill notes, “uncertainty is part of the knowledge machinery of scientific practice,” yet in the case of epigenetics an “epistemic ostentatiousness” has dogged research in this area (Pickersgill, 2016). Our concern is that, as scientific knowledge is a coproduction subject to social forces (Jasanoff, 2004), new research that seeks to understand preconception as an important window for intervention will continue to frame this in a way that is overly focused on female bodies, without the “epistemic modesty” (Pickersgill, 2016) that is perhaps necessary in what is a rapidly shifting area of research. Furthermore, the “temporal ambiguity” of the preconception period (Waggoner, 2013, p. 356) is amplified in the DOHaD framework, which postulates epigenetic mechanisms and a view of life stages as a series of “folds” rather than linear sequences (Warin et al., 2015; Mansfield, 2017; Pentecost and Cousins, 2017; Meloni, 2018). Despite the positive possibilities of the kinds of relationality that the “postgenomic body” might afford, the pragmatic translation of the science most often reverts to a foregrounding of the maternal body and the female reproductive body as sites of intervention (Meloni, 2018; Pentecost and Ross, 2019).

## Anticipating New Norms

The expanded concern about preconception care in biomedicine is one lens through which to understand how norms that challenge nature–society binaries in the conceptualization of body–environment interactions are being re-defined (Lock, 2017). A global public health vision of the “preconception period” has been reinvigorated and expanded by scientific findings that link preconception to later health outcomes. This has important implications for the formalization and consolidation of the idea of a preconception “developmental period” in which direct adverse effects can be established between behaviors and health trajectories (Kobor and Weinberg, 2011). These implications include questions about the envisaged boundaries of the normal and the pathological that such developments might herald. How *should* “preconception” be defined, and how is it defined or experienced in ordinary life? What does it mean to plan, or to be unplanned? Does renewed attention to preconception care offer up a new norm around a *planned* conception as a *good* conception? At the collective level: how are sub-populations, such as pregnant women and potentially reproductive women, perceived to be so permeable to the power of places and experiences to be managed? If preconception is “before the beginning,” as Stephenson et al. (2018) put it, when is the beginning? And finally how do we include in this model the stochastic and contingent nature of biological development rather than simplistic metaphors of programming or early-life determination (Davey Smith, 2011)?

These are instructive questions, inquiring as they do about how we conceive of new notions of life and of the boundaries of life and death (Han and Das, 2015). It is in trying to answer these questions that a return to Canguilhem's seminal work, *The Normal and the Pathological* is useful. In this text, he states that the “normal or physiological state is no longer simply a disposition which can be revealed and explained as a fact but a manifestation of an attachment to some value” (Canguilhem, 1991 [1978]: p. 57). In this formulation, “health is a margin of tolerance for the inconstancies of the environment” (p. 197), and, conversely, “disease is characterized by the fact that it is a reduction in the margin of tolerance for the environment's inconstancies” (p. 199). So, he continues, “[Wom]an feels in good health—which is health itself—only when [s]he feels more than normal—that is, adapted to the environment and its demands—but normative, capable of following new norms” (p. 200). Put more simply, “[wo]man is healthy insofar as [s]he is normative relative to the fluctuations of h[er] environment” (p. 228).

In this case, what happens to our understandings of health, the normal and the pathological, when an individual of reproductive potential is compelled to engage in behaviors thought to maintain the “correct” environment in anticipation of conception? *Individuality*, as an unsolved problem in Canguilhem's work (Han and Das, 2015), becomes further unsettled. As Susanne Bauer has argued, a life course approach incorporating public health genomics “folds individual and population health into each other” predicting individual risk across the life course with computational risk modeling that troubles public health approaches to the differences between individuals and groups (Bauer, 2013). Drawing on the Deleuzian notion of “dividual” subjectivity (Deleuze, 1992), she argues that this new epidemiological approach performs “in/dividuals as data points moving from fetal development and early childhood to adult life.” What emerges then is a form of “metabolic individuality”: “a reassemblage of genetic disposition and exposure, including epigenetic transmissions of exposure of previous generations” (Bauer, 2013, p. 525). We could say that Canguilhem anticipates this (dividual) norm located across bodies and time.

While life course models of intervention are linear and rooted in individual bodies, epigenetic models disrupt this linearity, offering repeated metaphors of “scaffolding” or “Russian dolls”, i.e., of life enveloped within itself (Warin et al., 2015; Meloni, 2018). Social scientists return frequently to describe this in terms of strata or folds (Warin et al., 2015; Mansfield, 2017; Pentecost and Cousins, 2017). As Lamoreaux (2016) argues, epigenetic research practices appear to contest individualization, while at the same time these discourses are translated into an intensified call for individual behavioral change across an expanded window of opportunity to affect future health outcomes. Intervening “preconception” exemplifies this logic. This constitutes, in our view, an important shift in the reframing of preconception as a state that is layered in time as opposed to a discrete, identifiable period before a pregnancy.

This implies a multi-layered temporal dimension to life's value. As Canguilhem notes, “the techniques of collective hygiene which tend to prolong human life, or the habits of negligence which result in shortening it, depending on the value attached to life in a given society, are in the end a value judgment expressed in the abstract number which is the average human life span” Canguilhem (1991, p. 161). Extended programs of preconception care radically expand “the techniques of collective hygiene” that optimize future health. Metrics that might capture a nation's preconception health status express a value attached to that future health. In Canguilhem's terms, this debate is one of relationship between organism and milieu, and one that is clearly socio-political: “recognition of the milieu's determining action has a political social impact: it authorizes man's unlimited action on himself via the intermediary of the milieu” (Canguilhem, 2008, p. 115). Norms, in this sense, now have an added temporal dimension. As Anderson (2010) and Adams et al. (2009) have argued, *anticipation* and *anticipatory action* have become a defining feature of the governance of life in the twenty-first century, and these anticipatory practices are frequently gendered (Adams et al., 2009). “Girlhood,” a central target of development logics since the 1990s, reflects a politics of temporality that is now mirrored in the increased public health concern with preconception.

The potential for heightened social and medical surveillance of bodies and individual reproductive decisions seems obvious, and is indeed reflected in reports such as the recent proposal for monitoring core metrics of preconception health in the UK, published in *The Lancet* (Stephenson et al., 2019). In this article, the authors argue for a “dual intervention strategy at both the public health level (e.g., by improving the food environment) and at the individual level (e.g., by better identification of those planning a pregnancy who would benefit from support to optimize health before conception) in order to raise awareness of preconception health and to *normalize the notion of planning and preparing for pregnancy*” (Stephenson et al., 2019: 2,262, our emphasis). The preconception population becomes the key operator for the implementation of a capillary form of biopower. It is the ambiguous banality of everyday life that matters for (epi)genomic health, where every action and lifestyle may have a direct impact on the integrity of the epigenome for present and future generations. At a population level, strategies include food fortification with folate, embedding preconception health in school curricula and in other policies related to maternal and child health, and broadly improving food environments (Stephenson et al., 2018. At the individual level, proposed strategies include “normalizing conversations about pregnancy intention” during clinic visits, providing online tools to promote behavior change, and training health care workers about preconception care Stephenson et al., 2019. This is more than detecting the presence of a faulty gene or highlighting risky alleles that may render the individual particularly susceptible to disease in a given environment. It is the emergence of a discourse of everyday self-regulation and anxious vigilance that resonates with contemporary neoliberal trends (Lupton, 2012) as well as ancient modalities of ascetic control of the female body (Kukla, 2005; Meloni, 2019). While interventions may be wrapped up in a rhetoric of benign aspiration, effectiveness, and optimization (Guthman and Mansfield, 2013; Waggoner, 2017; Wastell and White, 2017; Pentecost and Ross, 2019), there is also potential for a deep personalization and moralization of what can be found in “abnormal” epigenomic markers (Meloni, 2019; cf. Dupras et al., 2019).

In this vein, “preconception care” aligns with the contemporary trend toward a continuous analysis of the personal risks and benefits associated with different choices and environments, and predictive rather than reactive medicine based on gathering large amounts of data from patients, even when they are well (Prainsack, 2018). Preconception care, in the expanded public health perspective now advocated, directs populations to engage in the continuous management of potentially adverse or beneficial exposures. Thus, if public health will inevitably fail to ensure that all pregnancies are “planned” (in an older, episodic logic), then strategies for continuous attention to preconception health, as described in the *Lancet* series, is seen as one way to overcome this.

It is this formulation that truly collapses the distinction between when one might feel unwell or “in good health,” or in Canguilhem's terms, “more than normal.” Such a formulation enfolds the potential for a new form of determinism that erases the very framework of bodily openness and potentiality that epigenetics is supposed to promote. Instead of mitigating against reductionism, such a view challenges Canguilhem's notion that life and health are contingent and that “biological norms should always be interpreted on an individual basis” (Gayon, 1998, p. 314). Contingency, individuality, and the stochastic nature of development are in fact replaced by a thought-style that posits strict causal links between (past or present) toxic environments and (present or future) deleterious phenotypic outcomes (Davey Smith, 2011). Paradoxically, the possibilities that the postgenomic era might offer for recognizing life as a process are subverted and undermined by the assumption of a stable and objective form of time, unshaped by its subject.

## Conclusion

As Nikolas Rose noted in 1998, new genetic technologies at that time offered “a radical revision in the very notion of corporeality and a revised spatialization and territorialization of living processes” (p. 161). As we have argued, contemporary logics of preconception intervention are buttressed by an added revision of the temporalities of life and care. In an age of evidence-based practice where evidence justifies and instantiates new norms, close attention to knowledge (co)production clearly remains an important task for feminist technoscience. We should, *pace* Canguilhem, persist in tracing the historical epistemologies (Daston, 1994) of these phenomena to eludicate “the epistemological field that allows for the production of what counts for knowledge at any given moment, and which accords salience to particular categories, divisions, classifications, relations and identities” [Poovey, 1995: 3, cited by Rose (1998)].

While twentieth-century genetics reconfigured prospective parents with a duty for *biomedical* rather than *lifestyle* intervention, the notion of a porosity of genetic expression to *the direct effects* of social experience (nutrition, stress, etc.) may undermine this assumption. With its combination of randomness and unavailability to social intervention, genetic determinism had an unintended but corrosive effect on the idea that parents may directly shape with their behavior intergenerational health trajectories. The logic of preconception challenges this disconnection. As such, it has the potential to expand ideas of the normal and normativity way beyond medical genetics, and revive frameworks that typically connected health to moral disciplining, particularly in the case of women as a special site of biological impressionability. As we have noted, there are three potential implications of this revival for individual and population health. First, the further normalization of individual responsibility of women for the health outcomes of their potential offspring. Second, the potential extension of this to cast both men and women from a potentially indefinite time in their lives as guardians of future generations' health. Third, the establishment of new and expanded metrics to measure the preconception health of populations with implications for the government of conduct and risk. As a site of renewed interest and intervention, “preconception” will require attention given the potential impacts on reproductive rights, gender roles and ideas of norms, normativity and the normalization of life.

## Data Availability Statement

The datasets generated for this study are available on request to the corresponding author.

## Author Contributions

MP conceived of an initial framework for the manuscript. MP and MM contributed equally to the writing of the manuscript.

## Conflict of Interest

The authors declare that the research was conducted in the absence of any commercial or financial relationships that could be construed as a potential conflict of interest. The reviewer LC declared a past co-authorship with one of the author MM to the handling editor.

## Footnotes

^1^We thank reviewer 2 for suggesting this important point.

^2^We say a “peculiar articulation” because it is important to note that other approaches in DOHaD, or even in “epigenetic epidemiology” (for instance, Waterland and Michels, 2007) are not really close to or compatible with a life course perspective. It goes beyond the scope of our article to fully cover the complex and diverse positions within the DOHaD constellation, or just within David Barker's work whose shifts and re-conceptualizations have been partly covered in other publications (see Warin et al., 2011).
